# The Impact of Internet Development on Urban Eco-Efficiency—A Quasi-Natural Experiment of “Broadband China” Pilot Policy

**DOI:** 10.3390/ijerph19031363

**Published:** 2022-01-26

**Authors:** Xiaoying Zhong, Guanghai Liu, Peng Chen, Kaili Ke, Ruhe Xie

**Affiliations:** 1 School of Economics and Statistics, Guangzhou University, Guangzhou 510006, China; zhongxy@nfu.edu.cn; 2 Business School, Nanfang College Guangzhou, Guangzhou 510970, China; 3 School of Management, Guangzhou University, Guangzhou 510006, China; rhxie@gzhu.edu.cn; 4 Industry and Planning Research Institute, China Academy of Information and Communications Technology, Guangzhou 510030, China; chenpeng1@caict.ac.cn; 5 School of Economics and Management, Southwest Jiaotong University, Chengdu 610031, China; kaili2021@my.swjtu.edu.cn

**Keywords:** Internet, eco-efficiency, “Broadband China” pilot policy, difference-in-difference method

## Abstract

Based on the panel data of 285 prefecture-level cities and above in China from 2005 to 2019, this paper takes the “Broadband China” pilot policy as a quasi-natural experiment and evaluates the impact of Internet development on urban eco-efficiency (symbolized by the “Broadband China” policy) by constructing multi-period difference-in-difference (DID) and spatial DID models. Results show that: the “Broadband China” pilot policy significantly improves the urban eco-efficiency: the eco-efficiency in pilot cities is about 16.8% higher than that in other cities. The results remain consistent after testing for robustness, including using estimation methods, excluding the sample of key cities, changing core explanatory variables, and introducing instrumental variables. Next, the influence of the “Broadband China” pilot policy on eco-efficiency is characterized by significant regional heterogeneity: Internet development significantly improves the eco-efficiency in the central, eastern and northeastern regions that are economically more developed and not resource-dependent. In contrast, this effect is not obvious in the western region that is economically less developed and resource-dependent. Moreover, the influencing mechanism of Internet development on eco-efficiency suggests that the “Broadband China” strategy boosts urban eco-efficiency by increasing the Internet penetration rate, improving technological innovation capacity, and upgrading the industrial structure. In addition, results from the spatial DID models indicate that the “Broadband China” pilot policy improves the eco-efficiency in local cities and significantly enhances that in neighboring cities. Based on this, this paper puts forward some suggestions regarding promoting new network infrastructure construction and differentiating development policies to fit local conditions.

## 1. Introduction

Humankind has created more material wealth in the 21st century than in the entire previous history. The same applies to the development of China. With its abundant natural resources and inexpensive labor, China has created a series of growth miracles since its economic reform and opening-up 40 years ago [1]. Nevertheless, when we review growth patterns over the past years, it is easy to notice that rapid growths were mainly due to the conquest of natural force, adoption of machines, application of chemistry in industry and agriculture and the reclamation of land [2]. Issues such as energy depletion, ecological degradation and increased environmental conflicts have risen. For many years, economic growth has come at the expense of environmental quality; however, global consensus on climate change, along with a shift to a green, low-carbon, resource-efficient economy, is emerging. Meanwhile, the rise of the Internet increases the chances of transforming the economic development model and promoting sustainable development, alleviating environmental pressure to a certain extent [3]. On the one hand, the Internet can drive the rapid development of new business models such as the digital economy and information technology application while eliminating lagged sectors that consume too much energy and cause severe pollution, improving the overall energy efficiency in key sectors and the eco-efficiency of the environment. On the other hand, Internet technologies can break time and space constraints in environmental governance, allowing us to conduct dynamic environmental monitoring, assess risks in real-time and offer timely feedback [4], and setting the stage for eliminating bottlenecks in resources and reshaping the ecosystem. Therefore, under the current targets of carbon peak and neutrality, it is crucial to understand the potential effect of the Internet on eco-efficiency accurately. This would help China implement the new development concept, build a new development pattern and promote the comprehensive green transformation of social and economic development.

China has entered a “new normal” (a new phase of economic development which emphasizes better quality growth). Current studies, both at home and abroad, have focused on economic growth effects [5,6,7], resource mismatch effects [8], production efficiency effects [9,10], industrial adjustment effects [11,12] and scientific and technological innovation effects [13,14]. Meanwhile, due to differences in geographic location, education, Internet access and information infrastructure, people’s ability to use computers and access the Internet varies greatly [15]. Whether the advancement of the Internet creates the digital divide has also been the center of attention for many scholars [16,17]. Regarding measures of Internet development, most studies have focused on Internet access and proliferation; therefore, have chosen a single indicator to measure Internet development, including the percentage of Internet users (or netizens) in residents live in one area at year-end [8], the per capita number of Internet users [18] and the Internet penetration rate [19,20]. In addition, another common approach to measure Internet development is to build an evaluation system by incorporating other factors such as infrastructure, macro environment, business applications and information resources [21,22,23].

The idea of eco-efficiency was first introduced by German scholars Schaltegger and Sturm [24]. The definition of eco-efficiency is not conclusive yet, but the basic idea is roughly the same: to obtain a larger economic output with smaller resources and environmental inputs [25]. The academic research on eco-efficiency has mainly focused on spatial scales at the national [26,27], provincial [28], and urban level [29,30]. There are three aspects: first, measurement methods of eco-efficiency. Widely adopted methods include using ratios [31,32], analytic hierarchy process [33], stochastic frontier analysis (SFA) [34,35], Data Envelopment Analysis (DEA) [36,37], Search Engine Brand Management (SBM) model [38]. Second, the temporal and spatial analysis of eco-efficiency. Most scholars have analyzed the spatial-temporal characteristics of the overall eco-efficiency in China, and it is generally agreed that despite the narrowing disparities in eco-efficiency between cities, the eastern region has the highest eco-efficiency [39,40]. Third, influencing factors of eco-efficiency. Scholars have analyzed influencing factors of eco-efficiency from many aspects. Current important factors include urban economic growth [40], population agglomeration [41], industry structural upgrading [42], resource utilization efficiency [43] and technological progress [44].

With the rise and advancement of the Internet, scholars at home and abroad have paid increasing attention to the role of the Internet in environmental governance, but relevant literature is limited. However, it is clear that Internet development has a complex effect on the ecological environment. On the one hand, by virtue of the technology itself, the Internet can promote information disclosure and facilitate information sharing, ultimately reducing environmental pollution and serving as a tool to save energy and reduce carbon emission [45]. Furthermore, Internet development or technological progress can naturally lower the per capita carbon emissions [46], reduce air pollution levels [47] and improve the urban environmental quality [3,48]. On the other hand, a few scholars have also suggested that Internet development works against the protection and governance of the ecological environment. In the long run, Internet development would increase carbon emissions [49]. Avom et al. [50] have analyzed 21 sub-Saharan African nations and found that the use of information and communication technologies (ICT), as measured by the penetration rate of mobile phones and the Internet, significantly boosts carbon dioxide emissions. With the gradual deepening of research, a growing number of scholars have agreed that the impact of Internet development on carbon emissions varies depending on the research method, region and development stage. Ref [51] found that ICT could have an inhibitory effect on carbon dioxide emissions once a threshold level of ICT development is achieved [52]. There is an inverted U-shaped relationship between the two.

Previous studies have mostly been limited to the impact of Internet development (or information technology) on environmental pollution (or carbon emissions). Few studies have investigated the effect of Internet development on urban eco-efficiency and its mechanism. In addition, when measuring the level of Internet development, previous studies mainly used indicators such as the number of web pages, domains and websites, or built an evaluation index system. These measures are deemed subjective. Some studies also treated innovative cities [42] or smart cities [53] as quasi-natural experiments (Natural experiment refers to an experimental research method in which individuals (or clusters of individuals) are exposed to the experimental or controlled conditions determined by natural or other factors that are not controlled by the observers. A quasi-natural experiment shares some similarities with a true experiment as it enables the researchers to control the research subjects by allowing for certain manipulation in a natural setting. The difference is that, unlike a real experimental design, a quasi-natural experiment lacks the element of random assignment to treatment or control group and is deemed less rigorous. In general, natural experiments are completely random. Quasi-natural experiments, by contrast, are not completely randomized experiments—they are approximations to completely randomized experiments.) with boundaries exceeding the extension of the Internet. Therefore, based on existing research, this paper takes the “Broadband China” pilot policy as a quasi-natural experiment to measure Internet development. Using a dataset of 285 prefecture-level and above cities in China from 2005 to 2019, this paper empirically tests the impact of Internet development, symbolized by the “Broadband China” policy, on the urban eco-efficiency by constructing multi-period DID models. The basic idea of the DID method is to compare the differences between the control group and the treatment group before and after the implementation of the policy, so as to reflect the impact of the “Broadband China” policy on ecological efficiency. 

Compared with existing research, this paper provides innovative insights in the following aspects: first, it makes up for the inadequacy of research on analyzing the impact of Internet development on urban eco-efficiency from the perspective of new infrastructure. In contrast to previous research on carbon emissions and environmental pollution, this paper takes eco-efficiency as the starting point, which would better reflect the comprehensive influence of the Internet on green, low-carbon, high-quality development. Second, using the exogenous policy of “Broadband China” in its identification strategy, this paper examines the impact of pilot policies on eco-efficiency by constructing multi-period DID methods. This method could be more objective than the existing literature methods (which use a single indicator or an evaluation index system). At the same time, this paper further analyzes the spatial spillover effects of the policy by adopting spatial DID models. Third, using technological innovation and industrial structure upgrading as mediators and constructing a mediating effect model, this paper investigates the influencing mechanism of the “Broadband China” pilot policy on eco-efficiency.

## 2. Theoretical Mechanism and Research Hypothesis

### 2.1. “Broadband China” Pilot Policy Background

As the Industrial Revolution advances, computer and information technology have developed rapidly. Science and technology have gradually become essential indicators to assess a country’s economic progress. In particular, network infrastructure, the basis for science and technology, has gradually attracted wide attention. However, China’s netizen population and the Internet penetration rate still lagged behind developed countries such as European countries and the United States. To effectively address this issue, China introduced the “Broadband China” strategy at the National Industry and Information Work Conference 2011, aiming to boost Internet speed and cut prices in a phased, effective approach. A total of 120 cities was selected as “Broadband China” pilot cities (clusters) in three patches in 2014, 2015 and 2016. After being selected as a pilot city (cluster), the city strives to boost broadband network speed, expand network coverage and increase the scale of broadband users with a goal to achieve full coverage for broadband access in urban and rural areas by 2020. It is foreseeable that the “Broadband China” pilot policy would improve Internet development in cities directly and facilitate local information transmission and knowledge diffusion. Meanwhile, the selection of “Broadband China” pilot cities is not logically influenced by the conditions of the local ecological environment. This would address reverse causality issues in these policies, it also provides a foundation for this paper to analyze the political effects of the Internet.

### 2.2. The Direct Impact of the “Broadband China” Strategy on Eco-Efficiency

Compared with traditional infrastructure, the Internet, as an essential foundation for technological development and innovation infrastructure, plays a significant role in increasing resource efficiency, reducing energy consumption and improving pollution control measures [54]. From a technology-driven perspective, the Internet has been the main driving force in improving eco-efficiency [53,55]. On the one hand, the Internet has boosted the development and popularization of information technology: the use of advanced technologies such as big data, cloud computing and the Internet of Things can reform existing polluting businesses, accelerate the transformation of scientific and technological achievements like clean energy, improve the efficiency of scientific and technological innovation and reduce the pressure on the ecological environment. On the other hand, the Internet can promote the introduction and popularization of advanced production and ecological technology by lowering the dissemination cost for advanced technology. It can also reduce enterprise energy consumption, thus facilitating the transition to cleaner production and increasing eco-efficiency [56]. From the perspective of economic structure transformation, achieving better coordination of the Internet and industrial management by integrating the former into the latter can improve eco-efficiency. On the one hand, the Internet can reduce transaction costs and information loss during the transition while improving resource utilization efficiency. It can also facilitate the emergence of new business forms and new models such as the digital economy and virtual economy, optimize the industrial structure and generate new economic forms. On the other hand, the Internet can eliminate lagged, high-polluting industries and adjust the industrial structure, raising the urban eco-efficiency [48]. From the perspective of upgrading production patterns, building broadband networks can help enterprises to accelerate network deployments and thus force traditional sectors to transform and upgrade. This would enable manufacturers to remodel their manufacturing process and innovate their business, driving the transition from traditional, extensive to intensive production modes, promoting clean production processes and enhancing eco-efficiency. From the perspective of environmental governance, the Internet can enhance environmental governance and enable a more effective joint prevention and control of the environment [57]. On the one hand, Internet technology changes the patterns in environmental governance, namely, from passive to forward-looking, from extensive to refined, from fragmentation to integrated and grid-based, bringing innovations to the concept of environmental governance. On the other hand, the Internet breaks information barriers and builds a green, interactive bridge connecting government, enterprises and the public [58]. This would effectively raise public awareness in environmental protection, create a social atmosphere that focuses on being eco-friendly, achieve transparency in environmental information and involve citizens in environmental co-governance. From the perspective of resource optimization allocation, the Internet boosts the overall level of information infrastructure in cities, reduces misallocation of resources, promotes the coupling and coordination between urban resource and environment carrying capacity, raises energy utilization efficiency and optimizes resource allocation, thus furthering green, smart, coordinated development of the region.

Based on the above analysis, this paper proposes:

**Hypothesis** **1** **(H1).** *The “Broadband China” pilot policy enhances urban eco-efficiency.*

### 2.3. The Indirect Impact Mechanism of the “Broadband China” Pilot Policy on Eco-Efficiency

1. “Broadband China” pilot policy affects eco-efficiency by increasing Internet penetration rate

The “Broadband China” pilot policy puts forward several specific goals in the field of the Internet. For example, China strives to achieve fiber-to-the-home broadband networks in cities and fiber-to-the-village coverage in rural areas by 2015, thus enhancing broadband access capability in cities and rural areas. The selected cities should be in a leading position regarding broadband access capability and broadband penetration rate. It can be expected that areas where the “Broadband China” strategy has been implemented would see significant improvements in the network infrastructure. These goals would force governments to invest more in infrastructure in a short time, which would directly drive the Internet penetration rate up. The rise of the Internet, along with its rising penetration, continues to increase the eco-efficiency of cities through many ways, such as technology, economic structure, production methods, environmental governance and resource allocation.

2. “Broadband China” pilot policy affects eco-efficiency by promoting industrial structure upgrading

The boosting effect of the “Broadband China” pilot policy on industrial structure upgrading is mainly reflected in two aspects: promoting new industries and new forms of business and transforming traditional industries. On the one hand, the “Broadband China” pilot policy accelerates industrial structure upgrading. The “Broadband China” pilot policy promotes the construction of network infrastructure that primarily supports the Internet. First, with sound network infrastructure in place, new economic growth models such as new forms of business, new types and new models have emerged. This would assist the progress of emerging industries such as information technology, cloud computing, mobile Internet and the Internet of Things, drive industrial upgrading and contribute to the optimization of industrial structure. Next, technological innovation helps to upgrade traditional production equipment. Hence, traditional industries would be immersed with new technology and knowledge, which would accelerate their integration with information technology, forcing them to transform into high-tech, high-efficiency and low-polluting industries and letting go of businesses and modules that are harmful to the environment. In addition, the development of network infrastructure has also enabled and triggered the evolution of sharing economy, which offers exact matching between supply and demand for traditional industries [59], driving the upgrading and transformation of traditional industrial structures. On the other hand, industrial structure upgrading increases eco-efficiency through division of labor and demonstration effects. First, specialization and division of labor in society arose from industrial structure upgrading. The higher the level of labor specialization, the more conducive to saving production costs, improving production efficiency and achieving cleaner production. The economies of scale and structural dividends arising from this process continue to improve the ecological effect. Next, the ongoing upgrades and innovations of existing products and technologies also help reduce emissions and protect the environment, thus enhancing the eco-efficiency of a city. Additionally, along with the upgrading of the regional industrial structure, this new development model would act as an example for other regions. This would help relevant enterprises increase manufacturing efficiency and realize green manufacturing, thus ultimately improving the overall eco-efficiency of society.

3. “Broadband China” pilot policy affects eco-efficiency by improving technological innovation capacity

The “Broadband China” pilot policy is beneficial to improve urban technological innovation capacity. This is mainly reflected in the following two aspects: first, the network infrastructure has the ability to disseminate information across time and space, and the Internet is capable of accelerating cross-regional integration of innovative elements such as funds, talents and technology. This would lead to knowledge spillover effects and encourage the diffusion of the technological innovation systems characterized by manufacturing, learning, research and using. It also stimulates technological progress in manufacturing, energy-saving and environmental protection for enterprises. Second, the application of Internet information technology covers all aspects of business productions and operations. As network infrastructure improves, enterprises have gradually strengthened their broadband networking capability and their network-based process reengineering and business innovation capabilities. This would also help enterprises enhance the technological innovation capabilities of their research and development (R&D) and production departments [21]. On the other hand, the improvement of technological innovation capability is conducive to improving the total factor productivity (TFP) of cities. From the standpoint of the Solow model, technological progress creates constant growth in TFP. Hence, technological innovation, underpinned by knowledge of the entire society, is the main driving force of economic growth (Romer, 1990). Technological innovation has promoted manufacturing and energy-saving technological progress and pushed enterprises to replace traditional resource-intensive products with technology-intensive ones. This process would fundamentally boost enterprises’ resource utilization and manufacturing efficiency and significantly promote the green development of cities [60], thus increasing the urban eco-efficiency.

Therefore, this paper proposes:

**Hypothesis** **2** **(H2).** *The “Broadband China” pilot policy enhances urban eco-efficiency by increasing the Internet penetration rate in cities, upgrading industrial structure and improving technological innovation capacity.*

### 2.4. The Spatial Spillover Effects of the “Broadband China” Pilot on Eco-Efficiency

While the “Broadband China” pilot policy improves the Internet in local areas, it also radiates outwards, creating an “urban integration effect.” New technology has reduced time and space constraints through the efficient transmission of data and accelerated inter-regional information sharing, knowledge accumulation and technology diffusion. It also enhances the breadth and depth of linkages in inter-regional economic activities and produces positive spillover effects [61]. Yilmaz et al. [62] have noticed the spatial spillover effect of informatization by conducting an empirical test using panel data of 48 states in the United States. With the emergence of new economic geography, Yilmaz et al. [63] has empirically confirmed network infrastructure’s spatial agglomeration and spillover effects. Research in China [64,65] has also supported the conclusion that the Internet has spatial spillovers effects. Guo and Chen [18] have drawn upon endogenous growth model and applied dynamics optimization analysis to clarify the Internet’s logical relationship and influence mechanism on environmental quality. Using panel data of 285 prefecture-level cities in China from 2005 to 2019, their analysis indicates that the Internet has significant negative direct effects and negative spillover effects on environmental pollution emissions. Moreover, according to the growth pole theory [66], economic development is not uniform over a region, but first takes place around a specific pole—due to differences in the eco-efficiency among cities—and then produces diffusion effects through various channels [67]. The development of network infrastructure has promoted this diffusion effect. Integrated information facilities can enhance market information symmetry, making advanced manufacturing technology and concepts more accessible to enterprises [68]. The development of networking technologies would drive technological advancement for enterprises and induce them into adopting clean production. This process will generate a learning effect on neighboring areas, which would increase the region’s overall eco-efficiency.

Thus, this paper proposes:

**Hypothesis** **3** **(H3).** *“Broadband China” pilot policy enhances the urban eco-efficiency in neighboring cities through spatial spillover effects.*

## 3. Methodology

### 3.1. Model

According to the classic IPAT model that measures human impact on the environment proposed by Holdren and Ehrlich [69], factoring influencing living environment include population, wealth and technologies. The general model is as follows:

(1) I=PAT

where I represents the condition of the environment, including resources and energy consumption and waste disposal, P represents population size, *A* stands for affluence, and T for the state of technology applied. This model is widely used in qualitative or quantitative research to analyze the relationship between environment, economy, population and technology. It also provides a theoretical framework for analyzing the influencing factors of environmental ecology. On this basis, Dietz and Rosa [70] have extended the IPAT model and proposed a stochastic version:

(2) $$I=aP_{i}^{b}A_{i}^{c}T_{i}^{d}e_{i}$$

where a, b, c, and d are parameters that need to be estimated, and e is the random error term. Taking the logarithm of both sides of Equation (2) gets the following reformulation:

(3) $$\ln I=\ln a+b\ln P_{i}+c\ln A_{i}+d\ln T_{i}$$

Considering that eco-efficiency aims to find a balance between environmental protection and economic development, this paper reformulates Equation (3) and uses EE (Eco-Efficiency) to replace I To accurately determine the causal relationship between Internet development and the eco-efficiency of the environment, this paper considers the “Broadband China” pilot policy implemented nationwide from 2014 as an exogenous shock. The Chinese government has approved 120 “Broadband China” pilot cities at year-end 2019. These randomized approvals can be considered a good quasi-natural experiment, providing a groundwork for DID models. Given that the pilot cities were approved in three batches, the paper draws on the multi-period DID approach proposed by Beck, Levine and Levkov [71], with cities that have not been selected in the pilot program being the control group and cities that have been selected in the pilot program being the experimental group. A two-way fixed-effect regression model is specified as follows:

(4) $$\ln EE_{it}=\alpha_{0}+\alpha \times did_{it}+\beta \times X_{it}+\mu_{i}+\eta_{t}+\varepsilon_{it}$$

(5) $$\mathrm{where}\;did_{it}=Time_{i}\times Group_{t}$$

In this formula, $EE_{it}$ denotes the eco-efficiency of city i in year t, and Time is a time dummy which takes on a value of 1 for the year when the “Broadband China” pilot begins and for the following years and 0 otherwise. Group is a dummy variable which is set to 1 for pilot cities and 0 for non-pilot cities. did is a dummy variable indicating whether the city has been selected as a “Broadband China” pilot city or not. If city i has been selected as a pilot city in year t, then this variable is set to 1 and otherwise to 0. α is the core coefficient of this paper, denoting the impact of the pilot policy on the urban eco-efficiency. If the “Broadband China” pilot policy increases urban eco-efficiency, then the variable α should be statistically significant positive. X represents a set of control variables containing population, affluence, energy consumption and industrial agglomeration. $\mu_{i}$ and $\eta_{t}$ are the individual and temporal fixed effects, respectively; and $\varepsilon_{it}$ is the random error term.

### 3.2. Description of Variables and Data

1. The predicted variable

Andersen and Petersen [72] have introduced the super-efficiency SBM model, and Tone [73] has further proposed the SBM model that incorporates undesirable outputs. The paper uses the SBM-Undesirable model to measure the eco-efficiency of 285 prefecture-level cities in China from 2005 to 2019. Table 1 lists the selected input/output indicators, where resource consumption is treated as input, economic growth as desirable output and environmental pollution as undesirable output. After assessing data availability, the article employs the non-oriented CRS- (constant returns to scale) based super-efficiency SBM model by using total electricity consumption, total water use and built-up area as inputs, the region’s gross domestic product (GDP) as the desired output, and the volume of industrial wastewater discharged, industrial SO_2_ emissions and industrial smoke (dust) emissions as undesirable outputs. The software used is MaxDEA 7.0 Pro. As for the dataset, this paper analyzes 285 prefecture-level cities in China, except six cities (Danzhou, Sansha, Chaohu (district was withdrawn and county established in 2011, through which historical districts became counties), Bijie, Tongren and Lhasa) because these cities have relatively more missing data.

2. The core explanatory variable

The paper treats the “Broadband China” pilot policy as a quasi-natural experiment and uses it to measure Internet development. It takes on a value of 1 for pilot cities and 0 for non-pilot cities and is set to 1 in the year of implementation and each subsequent year and 0 before the implementation. The paper obtains the list of “Broadband China” pilot cities from the website of the Ministry of Industry and Information Technology of China. The pilot list includes certain autonomous regions (for example, Wenshan Zhuang Miao Autonomous Prefecture), certain districts in municipalities (for example, Jiulongpo District and Beibei District in Chongqing), certain cities at the county-level (Yongcheng City in Shangqiu City, Henan Province, and Kunshan City in Suzhou City) and cities with serious data deficiencies (Linzhi City in Tibet Autonomous Region). After dropping these areas, the final research sample contains 108 cities in the experimental group and 177 cities in the control group.

3. The controlled variable

According to existing literature, given the heterogeneity among cities and the prevention of omitted variables, the paper also controls for other variables that might influence eco-efficiency. Other variables include economic development level (GDP), measured by the logarithm of the gross domestic product (year 2000 is used as the base year to calculate the average GDP index); population density (PEO), defined as the logarithm of the total population per square kilometer; energy intensity (EI), measured as the industrial SO_2_ emissions per unit of GDP; industrial agglomeration (LQ), expressed as the location entropy of the secondary industry, the formula is specified as $LQ_{ij}=(q_{ij}/q_{j})/(q_{i}/q)$, where $q_{ij}$ is the gross domestic product of the secondary industry of region j, $q_{j}$ is the GDP of region j, $q_{i}$ is the gross domestic product of the secondary industry at the national level, and q is the country’s GDP; foreign direct investment (FDI), indicated by the proportion of FDI in GDP, and the proportion of fiscal expenditure (GOV), measured as the ratio of government fiscal expenditure to the regional GDP, with US dollar to Chinese yuan exchange rate obtained from the official website of the People’s Bank of China.

The sample includes data of 285 cities at the prefecture-level and above in mainland China, except Hong Kong, Macao and Taiwan, from 2005 to 2019, given the availability of data. The relevant data are taken from various sources, such as the China City Statistical Yearbook, the Provincial Statistical Yearbooks and the China Statistical Yearbook for Regional Economy. In addition, there is a small portion of data missing and has been supplemented by using the mean substitution method and multiple imputation method. To smooth the data, this paper takes the logarithm of some of the explanatory variables.

### 3.3. Descriptive Statistics of Variables

The final sample contains data from 285 prefecture-level cities in China from 2005 to 2019. Table 2 displays the descriptive statistics of the variables used.

## 4. Impact of Internet Development on the Urban Eco-Efficiency

### 4.1. Parallel Trend Test

**Figure 1:**
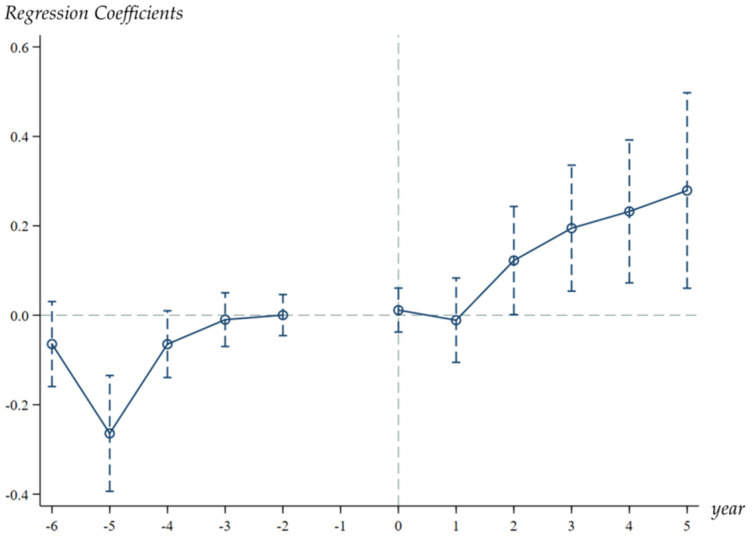
Test for parallel trends.

The premise of using the DID method to conduct policy evaluation is that the parallel trend assumption is met; that is, before the implementation of the pilot policy, the urban eco-efficiency should remain steady in both the experimental and the control group. Therefore, the paper employs the method proposed by [74,75,76]. It uses an event study framework to test for common trends in the DID approach. The following dynamic model is constructed:

(6) $$\ln EE_{it}=\alpha_{0}+\sum_{k=-6}^{3}\beta_{k}\times D_{i,t+k}+\alpha_{2}X_{it}+\mu_{i}+\eta_{t}+\varepsilon_{it}$$

where i and t represent city and year, respectively, $D_{i,t+k}$ is a dummy variable indicating whether the city has been selected as the “Broadband China” pilot city or not. A negative k denotes that it is k years before the year when the city becomes a pilot city, whereas a positive *k* means that the city has become a pilot city for k years $\beta_{k}$ is the key variable, representing the difference between the experimental group and the control group before or after k years. If k<−1 and $\beta_{k}$ variable is not significantly different from 0, then the test for parallel trends is passed. Figure 1 displays the coefficient of $\beta_{k}$ in the interval of −6≤k≤6 and its 95% confidence interval, showing the changes of $\beta_{k}$ in a more intuitive way. It can be observed that the estimated values of $\beta_{k}$ have remained fairly flat in the interval of −6≤k<−1, indicating that there is no significant difference between pilot cities and non-pilot cities before policy implementation. The estimated value of $\beta_{k}$ starts to rise significantly from k=0, which means that the “Broadband China” pilot policy has improved the eco-efficiency of pilot cities. Moreover, the results also suggest that the test for parallel trends is passed. Therefore, DID approach is a reasonable method to examine the impact of the “Broadband China” pilot policy on urban eco-efficiency.

### 4.2. Baseline Regression Results

The paper employs a two-way fixed effects model to evaluate the impact of Internet development, which is driven by the “Broadband China” pilot policy, on urban eco-efficiency. Table 3 presents the estimation results. Model 1 shows the estimation results of a model without any controlled variables, and Models 2–6 display the regression results of models with controlled variables introduced one by one. It is not difficult to find that the “Broadband China” pilot policy significantly increases the urban eco-efficiency after controlling for the individual and temporal fixed effects, regardless of whether controlled variables are added or not. The eco-efficiency of pilot cities increases 16.8% on average than that of their non-pilot peers, and the results are significant at a 1% confidence level; all other terms and conditions remain unchanged. We believe there are several reasons for this. First, the “Broadband China” pilot cities have put forward specific, quantifiable goals for network infrastructure construction. After a relatively short construction period, this would advance Internet development and facilitate the dissemination of technology and knowledge that uses the Internet as a medium, which helps introduce and popularize advanced manufacturing technologies and reduces energy usage in the manufacturing industry in cities. Meanwhile, Internet development can eliminate lagged, hand-made, high-polluting industries. The advance of AI and digital technology has led to emerging industries and thus promotes industrial structure upgrading in cities. Such activities have direct impacts on eco-efficiency.

With regard to the controlled variables, results show that the sample tends to give more stable estimation results with more controlled variables added, and the impact of GDP on urban eco-efficiency remains significantly positive. Generally speaking, people are inclined to have higher requirements for environmental quality as the economy grows. Thus more funds will be spent on environmental governance. Urban eco-efficiency and economic growth are complementary and mutually supportive. Energy intensity, measured as the industrial SO_2_ emissions per unit of GDP, is significantly negative at 1%, with a coefficient of −0.67. SO_2_ is a typical air pollutant and can reflect the effect of inter-regional pollution transmission [77]. Increased emissions of SO_2_ are considered of all harms but no benefit. Government spending also has a negative impact on eco-efficiency, with an estimated value of −0.62 and is significant at the 5%. This indicates that an increased proportion of fiscal expenditures is detrimental to improving eco-efficiency.

Moreover, the impacts of population density, industrial agglomeration and FID on the eco-efficiency are all positive, but none passes the significance test. Industrial agglomeration facilitates infrastructure sharing, and Internet development promotes the digital transformation of business, both of which help reduce carbon emissions, achieve carbon neutrality and improve the urban eco-efficiency. International trade has long been believed to affect pollution. The effects of trade on the environment have long been interpreted as follows: in the past, China was considered the world’s “pollution refuge” or “pollution haven” due to its policy of opening up to the outside world. However, as reform and opening up deepen, this situation has changed: China used to be the primary destination for the developed countries’ high-polluting, high-energy-consuming manufacturing sectors, whereas now, more and more high-value-added service sectors are making their ways to China. While China remains an integral part of the global value chain, the phenomenon of “pollution refuge” has eased.

### 4.3. Robustness Tests

The above results indicate that the “Broadband China” pilot policy contributes significantly to the urban eco-efficiency. To further verify the reliability and stability of these regression results, this paper conducts a series of robustness tests, including using different estimation methods, excluding samples of key cities and replacing core explanatory variables. By re-estimating the results with additional tests, it is suggested that the regression results of the multi-period DID models are robust.

1. Using different estimation methods

In addition to using two-way fixed effects regression methods for estimating, this paper also uses reg, areg and reghdfe [78,79,80], three common estimation commands for multi-period DID models, to run the regression. Table 4 presents the comparisons of results of these three different methods. These regression results are consistent with those of the two-way fixed effects model. The estimated values of did in these three regressions are 0.168, and remain significant at a 1% confidence level. The coefficients in these four commands are the same. Compared to the results of the fixed-effects model, only the standard score of the coefficients, the constant term and the goodness-of-fit measure ($R^{2}$) have changed. These results verify that these four methods are equivalent estimations.

2. Excluding samples of key cities

Sample data used in the paper is panel data of cities at the prefecture-level; however, there are four municipalities directly under the administration of the central government, namely Beijing, Tianjin, Shanghai and Chongqing. With large sizes and huge populations, these four cities play a leading role in China’s economic growth and are the regional economic and social development growth poles. Given the significant disparities in supporting policies and economic growth between these four cities and others, data of these four cities can be considered more of provincial-level. To ensure the robustness of the regression results, this paper, therefore, excludes the data of these four municipalities from the full sample and applies the two-way fixed effects model to re-examine the impact of the “Broadband China” pilot policy on the eco-efficiency. The results are shown in columns 1–2 of Table 5. After excluding samples of key cities, the coefficient of the variable representing the impact of the “Broadband China” pilot policy on the eco-efficiency changes slightly. Its value falls slightly, from 0.168 to 0.156, when controlled variables are added. However, it remains significant at a 1% confidence level, regardless of whether the controlled variables are added or not. In addition, the signs of other coefficients also remain largely the same.

3. Replacing a core explanatory variable

The Internet penetration rate is more representative in measuring the level of Internet development than traditional measures such as the number of webpages, domain names and websites. Based on the approach applied by Ye Chusheng et al. (2018) [81], this paper further uses the per capita number of Internet users as the core explanatory variable. This indicator, defined as the percentage of the population that are Internet broadband subscribers at year-end, is a good indicator of Internet coverage. This data is both continuous and randomly distributed. Based on this, this paper uses a panel model to test the robustness of the regression model. The estimated fixed and random effects results are shown in columns 3–6 of Table 5. Based on the results of the Hausman test, the fixed-effects model containing all variables is then selected. The estimation results show that the coefficients of Internet development are all significantly positive at 10%; that is, the estimation results are relatively more robust in the model with this replaced core explanatory variable.

### 4.4. Endogeneity

Endogeneity may exist between pilot policies and urban eco-efficiency. On the one hand, Internet development may improve the urban eco-efficiency by enhancing network penetration, upgrading industrial structure and improving technological innovation capability. On the other hand, cities with higher levels of eco-efficiency tend to have strong overall strength. With a better endowment of resources and a more robust economy, these cities attract the continuous inflows of external resources and become more capable of building network infrastructure, which would accelerate Internet development. Meanwhile, there are many factors that influence urban eco-efficiency. Hence, the controlled variables selected in this paper cannot avoid the omitted variable problem. Scientifically appropriate instrumental variables are among the most common methods to address the endogenous problem caused by reverse causality in regressions. A good instrumental variable usually has two important characteristics: first, it is highly correlated with the endogenous variable (which is the Internet development); second, this variable does not directly affect the predicted variable (which is the eco-efficiency); that is, the choice of instrumental variable needs to meet two criteria: relevance and exogeneity.

Given these considerations, this paper studies existing literature and adopts the idea of Kolko (2012) [5] by using the slope of terrain leans as an instrumental variable for the “Broadband China” pilot policy. On the one hand, in terms of relevance, the slope of terrain leans directly influences network infrastructure’s construction and operation costs: mountainous areas generally have lower network coverage and poorer network signal quality than plain areas. Therefore, in theory, the slope of local terrain would negatively impact Internet development. On the other hand, the slope of local terrain is a basic geographical characteristic and has no impact on eco-efficiency, thus meeting the exogenous requirements of instrumental variables. Hence this paper conducts the regression using the slope of local terrain as an instrumental variable. The regression results are shown in Table 6. The regression results of the first stage show that the slope of local terrain has a negative relationship with the “Broadband China” pilot policy and remains significant at 1%. This means that the slope of local terrain inhibits Internet development, which is consistent with previous theoretical analysis. The regression results of the second stage show that the relevant coefficient becomes closer to the baseline result after controlling for other variables. This suggests that the “Broadband China” pilot policy improves eco-efficiency.

## 5. Further Estimations

### 5.1. Heterogeneity

1. Heterogeneity of urban locations

From the perspective of urban locations, China’s economy presents strong spatial differences. There is a noticeable development gap among the eastern, central, western and northeastern regions. The construction of network infrastructure may have a different impact on eco-efficiency due to the city’s geographic location. Therefore, this paper further divides the 285 sample cities into four major regions (The eastern region includes 10 provinces (municipalities) namely Beijing, Tianjin, Hebei, Shanghai, Jiangsu, Zhejiang, Fujian, Shandong, Guangdong and Hainan; the central region includes 6 provinces of Shanxi, Anhui, Jiangxi, Henan, Hubei and Hunan; the western region includes 12 provinces (autonomous regions and municipalities), namely Sichuan, Guizhou, Yunnan, Tibet, Shaanxi, Gansu, Qinghai, Ningxia, Xinjiang, Guangxi, Chongqing and Inner Mongolia; the northeast region mainly includes 3 provinces of Liaoning, Jilin and Heilongjiang.) eastern, central, western and northeastern regions—to analyze the impact of Internet development on eco-efficiency. As a result, there are 87 cities in the eastern region, 80 in the central region, 84 in the western region and 34 in the northeastern region. Regression analysis is performed for each region, with results shown in Table 7. 

The results indicate that the coefficients of variables representing the “Broadband China” pilot policy are significant at 5%, suggesting that this pilot policy promotes eco-efficiency in the eastern, central and northeast regions. After controlling for other factors, the coefficients for these three regions are 0.260, 0.283, and 0.287. The coefficient in the western region is insignificant, standing at −0.086, suggesting that the “Broadband China” pilot policy has a negative but insignificant impact on the eco-efficiency in the western region. Based on the above results, it can be concluded that Internet development, driven by the “Broadband China” pilot policy, improves the ecological environment nationwide but has a greater impact in central and eastern regions. The reason could be that the central and eastern regions are endowed with natural resources and geographical advantages and have been equipped with a higher level of network infrastructure, whereas the environment in the western region is relatively more unpleasant. Its undulating terrain means a long infrastructure construction period. As a result, network infrastructure development is lagging and tends to be harmful to the ecological environment.

2. Heterogeneity of city scale

The heterogeneity of city scale means that there are big differences between larger cities and smaller cities in industrial structure, energy consumption and technological development, causing variations in the impact of the “Broadband China” pilot policy on the ecological environment. Thus, this paper conducts additional regression analysis by dividing the sample into economically developed and underdeveloped cities by whether the per capita GDP of each city in 2018 is higher than the national per capita GDP of the same year. The results are shown in Table 8. The results indicate that the “Broadband China” pilot policy significantly enhances eco-efficiency in developed cities, where its impact in underdeveloped cities is insignificant. Possible reasons could be that the developed cities have a sound basis for Internet development. The “Broadband China” pilot policy can promote resource allocation efficiency, technological advances and industrial structure optimization in large and medium-sized cities, thereby promoting the urban eco-efficiency. However, as underdeveloped cities lack the funds required to construct infrastructure and have a low level of technological advance, network infrastructure construction fails to produce desired policy outcomes in the short term. Therefore, a statistically insignificant causal relation is explainable.

3. Heterogeneity of resource dependence

Resource-based cities are the types of cities that exploit and process natural resources, such as minerals and forests in the region. The production and development of cities are closely related to resource exploitation and processing, which greatly influences eco-efficiency. This paper refers to the Sustainable Development Plan for National Resource-Based Cities (2013–2020) issued by the State Council and divides samples into two types: resource-based cities and non-resource-based cities. The number of cities in each type is 112 and 173. Regression results are shown in Table 8. The coefficient value of the variable representing the “Broadband China” pilot policy is 0.202, suggesting that the “Broadband China” pilot policy in non-resource-based cities has significantly promoted the urban eco-efficiency. However, this impact is insignificant for resource-based cities. Resource-exhausted cities have contributed to China’s economic growth in the early days of China, but its industrial development remains highly dependent on resources to date. In addition, these cities suffer simple industrial structure, excessive development intensity, low resource utilization and damaged ecological environment. Hence, it is hard for the development of the Internet, driven by the construction of network infrastructure, to produce significant policy effects in a short time. Generally speaking, non-resource-based cities tend to have a more complete and diversified industrial structure, making the “Broadband China” pilot policy easier to promote resource allocation efficiency, technology advancement and industrial structure upgrading, thereby enhancing urban ecology efficiency.

### 5.2. Influencing Mechanism Analysis

To verify whether Internet development can promote eco-efficiency through other mechanisms, this paper further tests the influencing mechanism of the two by constructing mediation models. The steps to test the mediating effect are as follows:

The first step: take eco-efficiency as the predicted variable and perform regression with the “Broadband China” pilot policy as the explanatory variable.

(7) $$\ln EE_{it}=\alpha_{0}+\alpha \times did_{it}+\beta_{j}X_{it}+\mu_{i}+\eta_{t}+\varepsilon_{it}$$

The second step: take mediators such as Internet penetration rate, industrial structure upgrading and technological innovation as the predicted variables and perform regression with the “Broadband China” pilot policy as the explanatory variable.

(8) $$M_{it}=\alpha_{0}+\alpha_{1}\times did_{it}+\beta_{j}X_{it}+\mu_{i}+\eta_{t}+\varepsilon_{it}$$

The third step: incorporate both the “Broadband China” pilot policy and mediators into the regression model and observe the impact of these two on total factor productivity growth.

(9) $$\ln EE_{it}=\alpha_{0}+\alpha_{2}\times did_{it}+\alpha_{3}M_{it}+\beta_{j}X_{it}+\mu_{i}+\eta_{t}+\varepsilon_{it}$$

where i represents city, t represents time, $EE_{it}$ denotes the condition of eco-efficiency for each city, did denotes the status of the “Broadband China” pilot policy in each city, and M is the mediator. Three mediators are used in this paper, namely Internet penetration rate, industrial structure upgrading and technological innovation. Internet penetration rate (NET) uses the per capita number of Internet users as the core explanatory variable and is measured as the percentage of the population that are Internet broadband subscribers at year-end. Industrial structure upgrading (INS) is measured by the ratio of tertiary industry output value to GDP, and technological innovation (INO) is represented by the number of patents authorized. According to the method for testing the mediation effect, the mediating effect exists if both α and $\alpha_{2}$ are significant and of different values.

In the regression results of the baseline model (as shown in Table 3) in the first step, the estimated value of did is significantly positive, suggesting that the “Broadband China” pilot policy significantly improves the eco-efficiency. Estimation results in the second and third steps where Internet penetration rate, industrial structure upgrading and technological innovation are used as mediators in the models are shown in Table 9. 

### 5.3. Spatial Spillover Effect Analysis

The National Measures for the Management of Construction of “Broadband China” Pilot Cities (Urban Clusters) implies that the “Broadband China” pilot city program should have a strong demonstration effect and leading role in similar regions across the country. Based on the previous theoretical analysis, the construction of information infrastructure has strong scale effects and network effects, generating a significant spatial spillover effect on “local-neighboring” economic activities. This would cause the causal effect identified by the traditional DID model to become invalid [82], and thus the model needs to be further expanded. To further test the spatial spillover effect of the “Broadband China” pilot policy on the improvement of eco-efficiency, this paper refers to the existing research [83] and constructs the spatial form of the DID model, that is, the spatial SDID model, based on Equation (4). The goal is to explore the spatial spillover effects of the “Broadband China” pilot policy on the eco-efficiency of cities in neighboring areas or with similar economic attributes.

(10) $$\ln EE_{it}=\alpha_{0}+\rho W\ln EE_{it}+\alpha \times did_{it}+\alpha_{1}Wdid_{it}+\beta \times X_{it}+WX_{it}\beta^{\prime }+\mu_{i}+\eta_{t}+{\left(I-\lambda W\right)}^{-1}\varepsilon_{it}$$

where W is the spatial weight matrix, representing the spatial relationships that exist among the sample data, ρ is the spatial autocorrelation coefficient of the predicted variable, $\beta^{\prime }$ is the spillover effect of other controlled variables, λ is the spatial autocorrelation coefficient of the random error term, and $\alpha_{1}$ is the spillover effect of the “Broadband China pilot policy. All other variables are the same as the baseline model. Equation (10) is the general form of SDID, which can be reformulated into three specific forms, namely spatial error model (SEM), spatial lag model (SLM), and spatial Durbin model (SDM), depending on which coefficient(s) is or are zero.

In choosing the spatial matrix, this paper mainly uses two spatial weight matrices to examine the spatial spillover effects of the “Broadband China” pilot policy on the eco-efficiency of neighboring and economically similar regions. The first matrix used is the geographical distance spatial weight matrix. This paper uses $d_{ij}$ to denote the geographical distance between cities, using the city longitude and latitude coordinates for this calculation, and applies the inverse distance as the spatial weight, defined as $W_{ij}=1/d_{ij},i\neq j$. The purpose is to explore the spillover effect of the pilot policy on geographically neighboring areas. The second matrix used is the economic distance spatial weight matrix. Referring to the practice of Lin Guangping (2005) [84], this paper builds an economic distance weighting matrix by using the inverse of the average difference between cities’ per capita GDP. The formula used is $W_{ij}=1/\left|{\bar{Y}}_{j}-{\bar{Y}}_{i}\right|(i\neq j)$, where Y represents the per capita GDP for each city. The purpose is to examine the spillover effects of pilot policy on economically similar regions.

Before employing the spatial DID model, this paper examines the spatial correlation of urban eco-efficiency. All the values of Moran’s I test for the sample cities are positive and significant at 1%, suggesting a significant spatial correlation between the eco-efficiencies of cities. Therefore, it is reasonable to consider spatial correlation based on the baseline model. The results of the Hausman test show that the fixed-effects model is more efficient than the random-effects model. Hence, when conducting spatial econometric analysis, this paper uses two-way fixed effects models.

Table 10 shows the estimated results of three spatial models using the geographical distance spatial weight matrix and the economic distance spatial weight matrix. It is found that, ρ or λ, the coefficient of the spatial lag term, is significantly positive at 1%, indicating that the “Broadband China” pilot policy has spillover effects and diffusion effects on the eco-efficiency and that the spillover effects are more significant when there are more neighboring cities. These results confirm Hypothesis 3. Moreover, the estimates of the core explanatory variable did are positive and significant at 1%, with values close to the results of the baseline model. This further proves Hypothesis 1, that is, the “Broadband China” pilot policy enhances the eco-efficiency of urban areas. In addition, the interaction term between the spatial weight matrix and the DID term (W.did) in the Spatial Durbin Model (SDM) under the geographical distance matrix is positive but statistically non-significant. Generally speaking, the “Broadband China” pilot policy has accelerated inter-regional knowledge and information technology transfer, flow and diffusion, and optimized the spatial allocation of information and resources that enable innovation, creating a strong demonstration effect on neighboring cities. However, the coefficient of the interaction term W.did under the economic distance spatial weight matrix is significantly negative at 1%. This indicates that the construction of information infrastructure between cities with similar economic attributes has a negative spatial spillover effect. With a limited supply of resources, being selected as pilot cities means that cities would have more financial resources and gain more support from relevant policies, which would plunder other economically similar, non-pilot cities’ information technology resources.

## 6. Conclusions and Prospects

To promote the comprehensive green transformation of social and economic development, China pledges to implement the new development philosophy and achieve “carbon peak” and “carbon neutral” with the help of Internet technology. Based on the panel data of 285 prefecture-level cities in China from 2005 to 2019, this paper evaluates the impact of Internet development on urban eco-efficiency (symbolized by the “Broadband China” policy) and its underlying mechanisms by constructing multi-period and spatial DID models via a quasi-natural experiment of the “broadband China” pilot policy. The conclusions are as follows: First, the results of the fixed-effects baseline model show that the “Broadband China” pilot policy significantly improves urban eco-efficiency. The results remain consistent after testing for robustness, including changing estimation methods, excluding the sample of key cities, replacing core explanatory variables and introducing instrumental variables. Next, the impact of the “broadband China” pilot policy on eco-efficiency has significant regional heterogeneity. Internet development significantly improves the eco-efficiency in the central, eastern and northeastern regions that are economically more developed and not resource-dependent. In contrast, the influence is not obvious in the western region that is economically less developed and resource-dependent. Furthermore, the analysis of the influencing mechanism of Internet development on eco-efficiency suggests that the “broadband China” strategy boosts urban eco-efficiency by increasing the urban Internet penetration rate, improving technological innovation capacity and upgrading the industrial structure. Finally, the spatial DID model results indicate that the “broadband China” pilot policy has a positive spillover effect on urban eco-efficiency and can improve the eco-efficiency in neighboring regions. Based on these findings, this paper proposes the following recommendations:

The new infrastructure initiative (symbolized by network infrastructure construction) plays an essential role in improving urban eco-efficiency. This provides a theoretical foundation and empirical evidence for a positive effect of Internet technology on high-quality and green economic development in the new era. Therefore, it is necessary to further implement the “Broadband China” strategy and advance the construction of digital facilities, strengthening the positive spillover effects of network infrastructure and unleashing the dividends of the digital economy. This would facilitate the internalization of new information technology, injecting new high-quality development momentum into cities and unleashing the benefits of boosting eco-efficiency and promoting green growth.

The results of heterogeneity analysis could serve as a useful reference for the direction of promoting network development. Regions should design their infrastructure policies according to their local conditions, resource endowments and economic development levels, give full play to key cities’ radiating and leading role, and improve flexibility and tolerance in implementing the “Broadband China” strategy. For underdeveloped and resource-dependent cities in the western region, it is crucial to accelerate the construction of local infrastructure, increase the support for technological innovation and form comparative advantages by utilizing their resources and introducing talents and technology. This would help these cities exploit the latecomer advantages and enable them to catch up with the developed cities, thanks to the increased eco-efficiency driven by Internet development.

Explore multiple ways for the “Broadband China” strategy to promote eco-efficiency, and thus maximize the impact of the “Broadband China” pilot policy. The first is to increase government spending on science and technology, use network infrastructure as a starting point and promote the co-construction and sharing of innovation platforms, thus enhancing the innovation capabilities of cities. The second is to strengthen the transformation and upgrading of the industrial structure by making full use of the Internet technology, further enhancing the driving effect of the Internet on industrial structure upgrading by transforming traditional sectors and promoting the emergence of large, new sectors. This would promote the coordinated development of ecology and economy and achieve “economy ecologization” and “ecological economicalization”, thus enhancing urban eco-efficiency.

There is still room for improvement in existing research. This paper evaluates the impact of Internet development on urban eco-efficiency by building a quasi-natural experiment using the “Broadband China” pilot policy. Although this paper’s conclusions are supported by rigorous analysis methods, more in-depth discussion is needed to examine whether the selection of policies can cover all aspects of Internet development and whether there are more appropriate policies. In addition, as countries, regions and city clusters are experiencing different levels of development, further research is also needed to determine whether this paper’s findings can be generalized. With the world entering the era of a digital economy where the growth of the digital economy is closely connected to the Internet, building high-tech smart cities has become one crucial path to solve urban problems such as resource scarcity and environmental pollution. More in-depth research can be carried out to determine the impact of the digital economy and smart cities on eco-efficiency.

## Figures and Tables

**Table 1:** Super-efficiency SBM model input/output indicators.

First-Level Indicator	Second-Level Indicator	Description
Resource consumption	Energy consumption	Total electricity consumption (KWh)
	Water consumption	Total water use (ten thousand ton)
	Land consumption	Built-up urban area (square kilometer)
Desirable output	GDP	Gross domestic product (100 million yuan)
Undesirable output	Wastewater discharged	The volume of industrial wastewater discharged (ten thousand ton)
	SO_2_ emissions	The volume of industrial SO_2_ emissions (ton)
	Smoke (dust) emissions	The volume of industrial smoke (dust) emissions (ton)

**Table 2:** Descriptive statistics of variables.

	Variable	Obs	Mean	Std. Dev.	Min	Max
Predicted variable	EE	4275	−1.6744	1.1154	−7.2811	0.2875
Explanatory variable	did	4275	0.1273	0.3333	0.0000	1.0000
Instrumental variable	IV	4275	0.6768	0.7542	0.0013	3.8138
Influential mechanism variable	NET	4275	0.1671	0.1827	0.0013	3.6635
	INS	4275	0.9191	0.5228	0.0943	9.4822
	INO	4275	0.3663	1.0161	0.0002	16.6609
Controlled variable	GDP	4275	15.8927	1.0135	12.8061	19.3892
	PEO	4275	5.7297	0.9161	1.5476	7.9227
	EI	4275	0.0639	0.1038	0.0000	1.5383
	LQ	4275	1.0844	0.2457	0.1914	1.9429
	FDI	4275	0.0188	0.0212	0.0000	0.3854
	GOV	4275	0.1764	0.1002	0.0000	1.4852

**Table 3:** Baseline regression results.

Variable	Model 1	Model 2	Model 3	Model 4	Model 5	Model 6
did	0.176 ***	0.176 ***	0.179 ***	0.179 ***	0.181 ***	0.168 ***
	(3.58)	(3.58)	(3.62)	(3.59)	(3.65)	(3.40)
GDP	0.657 ***	0.657 ***	0.644 ***	0.643 ***	0.646 ***	0.584 ***
	(6.23)	(6.23)	(6.22)	(5.29)	(5.29)	(4.70)
PEO		0.0166	0.0191	0.0192	0.0218	0.0177
		(0.19)	(0.22)	(0.22)	(0.25)	(0.21)
EI			−0.682 ***	−0.682 ***	−0.669 ***	−0.669 ***
			(−3.10)	(−3.10)	(−3.04)	(−3.07)
LQ				0.00121	−0.00562	−0.0139
				(0.01)	(−0.04)	(−0.11)
FDI					0.540	0.669
					(1.03)	(1.27)
GOV						−0.620 **
						(−2.51)
C	−11.84 ***	−11.93 ***	−11.64 ***	−11.63 ***	−11.70 ***	−10.65 ***
	(−7.42)	(−7.08)	(−7.06)	(−6.30)	(−6.29)	(−5.63)
Regional effect	YES	YES	YES	YES	YES	YES
Time effect	YES	YES	YES	YES	YES	YES
N	4275	4275	4275	4275	4275	4275
$R^{2}$	0.830	0.830	0.832	0.832	0.832	0.833

Note: ** *p* < 0.05, *** *p* < 0.01, Numbers in parentheses indicate z-scores.

**Table 4:** Estimation results of models using different multi-period DID methods.

Variable	Reg		Areg		Reghdfe	
	(1)	(2)	(3)	(4)	(5)	(6)
did	0.185 ***	0.168 ***	0.185 ***	0.168 ***	0.185 ***	0.168 ***
	(6.33)	(5.82)	(6.33)	(5.82)	(6.33)	(5.82)
GDP		0.584 ***		0.584 ***		0.584 ***
		(6.11)		(6.11)		(6.11)
PEO		0.0177		0.0177		0.0177
		(0.22)		(0.22)		(0.22)
EI		−0.669 ***		−0.669 ***		−0.669 ***
		(−4.27)		(−4.27)		(−4.27)
LQ		−0.0139		−0.0139		−0.0139
		(−0.15)		(−0.15)		(−0.15)
FDI		0.669		0.669		0.669
		(1.48)		(1.48)		(1.48)
GOV		−0.620 ***		−0.620 ***		−0.620 ***
		(−3.19)		(−3.19)		(−3.19)
C	−0.766 ***	−11.08 ***	−1.906 ***	−10.65 ***	−1.698 ***	−10.92 ***
	(−4.63)	(−6.31)	(−76.04)	(−7.25)	(−224.15)	(−7.10)
Regional effect	YES	YES	YES	YES	YES	YES
Time effect	YES	YES	YES	YES	YES	YES
N	4275	4275	4275	4275	4275	4275
$R^{2}$	0.870	0.876	0.870	0.876	0.870	0.876

Note: *** *p* < 0.01, Numbers in parentheses indicate z-scores.

**Table 5:** Estimation results of models after excluding samples of key cities or replacing a core explanatory variable.

Variable	Excluding Samples of Key Cities		Replacing a Core Explanatory Variable			
	(1)	(2)	(3)	(4)	(5)	(6)
did	0.171 ***	0.156 ***				
	(3.34)	(3.10)				
NET			1.343 ***	0.177 *	1.174 ***	0.123
			(5.64)	(1.76)	(6.10)	(0.88)
GDP		0.569 ***		0.669 ***		0.315 ***
		(4.51)		(9.60)		(8.16)
PEO		0.0149		0.0398		−0.0556
		(0.17)		(0.10)		(−1.17)
EI		−0.676 ***		−0.619 **		−1.364 ***
		(−3.09)		(−2.27)		(−4.00)
LQ		0.00457		0.0257		0.0260
		(0.04)		(0.18)		(0.23)
FDI		0.718		1.184		−1.898 **
		(1.34)		(1.40)		(−2.44)
GOV		−0.617 **		−1.966 ***		0.205
		(−2.51)		(−4.12)		(0.88)
C	−1.906 ***	−10.41 ***	−1.899 ***	−12.22 ***	−1.871 ***	−6.331 ***
	(−78.01)	(−5.44)	(−47.77)	(−6.06)	(−38.26)	(−10.88)
Regional effect	YES	YES	YES	YES	YES	YES
Time effect	YES	YES	YES	YES	YES	YES
*Model*	—	—	*FE*	*FE*	*RE*	*RE*
N	4215	4215	4275	4275	4275	4275
$R^{2}$	0.824	0.833	0.033	0.089	0.033	0.172

Note: * *p* < 0.1, ** *p* < 0.05, *** *p* < 0.01, Numbers in parentheses indicate z-scores.

**Table 6:** Estimation results of models with instrumental variables.

Variable	First Stage	Second Stage	
IV	−0.0079 ***		
	(−2.84)		
did		9.07	1.104 ***
		(1.16)	(2.89)
C	0.0938 ***	−2.515 ***	−2.099 ***
	(9.84)	(−3.64)	(3.78)
Controlled variable	YES	YES	YES
N	3705	3705	3705

Note: *** *p* < 0.01, Numbers in parentheses indicate z-scores.

**Table 7:** Estimation results of multi-period DID models by regions.

Variable	East		Central		West		Northeast	
	(1)	(2)	(3)	(4)	(5)	(6)	(7)	(8)
*did*	0.231 ***	0.260 ***	0.342 ***	0.283 ***	−0.0709	−0.0863	0.316 *	0.287 **
	(3.04)	(3.37)	(3.64)	(3.18)	(−0.73)	(−0.92)	(1.92)	(2.11)
*GDP*		0.924 ***		0.849 ***		0.543 **		0.227
		(4.18)		(2.97)		(2.56)		(0.63)
*PEO*		0.0463		0.0429		−0.174		−0.846
		(0.44)		(0.31)		(−0.55)		(−0.60)
*EI*		−4.47 ***		−1.74 ***		−0.206		−0.912
		(−4.80)		(−4.30)		(−1.14)		(−1.42)
*LQ*		−0.153		−0.0082		−0.0199		0.422
		(−0.65)		(−0.03)		(−0.09)		(1.52)
*FDI*		−0.624		−0.133		0.907 **		1.611
		(−0.46)		(−0.10)		(2.15)		(0.87)
*GOV*		−0.669 *		−1.883 *		−0.713 **		−1.379
		(−1.73)		(−1.72)		(−2.02)		(−1.54)
*C*	−1.65 ***	−15.9 ***	−1.99 ***	−14.5 ***	−1.96 ***	−8.82 **	−2.23 ***	−1.446
	(−32.9)	(−4.6)	(−55.6)	(−3.3)	(−42.0)	(−2.42)	(−33.2)	(−0.16)
Regional effect	YES	YES	YES	YES	YES	YES	YES	YES
Time effect	YES	YES	YES	YES	YES	YES	YES	YES
*N*	1305	1305	1200	1200	1260	1260	510	510
*R* ^2^	0.841	0.855	0.850	0.867	0.831	0.840	0.766	0.738

Note: * *p* < 0.1, ** *p* < 0.05, *** *p* < 0.01, Numbers in parentheses indicate z-scores.

**Table 8:** Regression results of cities with different urban capacity and resource dependence.

Variable	Urban Capacity				Resource Dependence			
	Developed City		Underdeveloped City		Resource-Based City		Non-Resource-Based City	
did	0.257 ***	0.254 ***	0.0427	0.0256	0.0525	0.0578	0.232 ***	0.202 ***
	(3.29)	(3.46)	(0.60)	(0.37)	(0.59)	(0.62)	(3.80)	(3.44)
GDP		0.934 ***		0.324 *		0.350 *		0.748 ***
		(4.51)		(1.84)		(1.83)		(4.39)
PEO		0.0821		−0.259 **		−0.0112		−0.0547
		(0.67)		(−2.07)		(−0.10)		(−0.49)
EI		−1.10 ***		−0.591 **		−1.19 ***		−0.338
		(−2.75)		(−2.51)		(−4.07)		(−1.25)
LQ		−0.345		0.243		−0.0038		−0.0117
		(−1.52)		(1.41)		(−0.02)		(−0.07)
FDI		1.813 *		0.304		−0.0481		1.689 *
		(1.75)		(0.63)		(−0.08)		(1.90)
GOV		−0.356 *		−0.705 **		−0.351		−0.882 **
		(−1.94)		(−2.16)		(−1.06)		(−2.22)
C	−1.94 ***	−16.7 ***	−1.89 ***	−5.27 **	−2.00 ***	−6.788 **	−1.84 ***	−12.9 ***
	(−46.3)	(−5.02)	(−64.9)	(−2.08)	(−50.7)	(−2.39)	(−60.8)	(−4.84)
Regional effect	YES	YES	YES	YES	YES	YES	YES	YES
Time effect	YES	YES	YES	YES	YES	YES	YES	YES
N	1515	1515	2760	2760	1680	1680	2595	2595
$R^{2}$	0.841	0.853	0.824	0.832	0.807	0.822	0.839	0.847

Note: * *p* < 0.1, ** *p* < 0.05, *** *p* < 0.01, Numbers in parentheses indicate z-scores.

**Table 9:** Estimation results of influencing mechanism.

Variable	*NET*		*INS*		*INO*	
	*M*	*EE*	*M*	*EE*	*M*	*EE*
did	0.0279 **	0.167 ***	0.0189 **	0.171 ***	0.447 ***	0.141 ***
	(2.21)	(3.36)	(2.3)	(3.49)	(3.80)	(2.85)
Mediator		0.0311		0.149 **		0.0619 ***
		(0.32)		(2.42)		(2.67)
C	0.552	−10.67 ***	−5.764	−9.797 ***	−7.163 *	−10.21 ***
	(1.39)	(−5.61)	(−1.44)	(−5.31)	(−1.87)	(−5.39)
Controlled variable	YES	YES	YES	YES	YES	YES
Regional effect	YES	YES	YES	YES	YES	YES
Time effect	YES	YES	YES	YES	YES	YES
N	4275	4275	4275	4275	4275	4275
$R^{2}$	0.484	0.833	0.760	0.833	0.258	0.834

Note: * *p* < 0.1, ** *p* < 0.05, *** *p* < 0.01, Numbers in parentheses indicate z-scores.

**Table 10:** Estimated results of spatial DID models with different weight matrices.

Variable	Geographical Distance Spatial Weight Matrix			Economic Distance Spatial Weight Matrix		
	SDM	SLM	SEM	SDM	SLM	SEM
ρ	0.974 ***	0.967 ***		0.915 ***	0.907 ***	
	(190.95)	(174.55)		(132.51)	(130.47)	
λ			0.972 ***			0.919 ***
			(191.78)			(135.96)
did	0.168 ***	0.135 ***	0.169 ***	0.184 ***	0.142 ***	0.190 ***
	(6.60)	(5.62)	(6.63)	(6.74)	(5.50)	(7.05)
W.did	0.137			−0.242 ***		
	(1.58)			(−4.21)		
Controlled variable	Controlled	Controlled	Controlled	Controlled	Controlled	Controlled
Regional effect	Controlled	Controlled	Controlled	Controlled	Controlled	Controlled
Time effect	Controlled	Controlled	Controlled	Controlled	Controlled	Controlled
*N*	4275	4275	4275	4275	4275	4275
*R* ^2^	0.001	0.151	0.067	0.035	0.042	0.072

Note: *** *p* < 0.01, Numbers in parentheses indicate z-scores.

## Data Availability

The relevant data are taken from various sources, such as the China City Statistical Yearbook, the Provincial Statistical Yearbooks and the China Statistical Yearbook for Regional Economy. In addition, there is a small portion of data missing and has been supplemented by using appropriate methods.
